# An Emergency Department Virtual Observation Unit Fall Prevention Program: A Pilot Acceptability Study

**DOI:** 10.7759/cureus.78624

**Published:** 2025-02-06

**Authors:** Kenji Numata, Abigail E Jones, Melissa A Meeker, Maura Kennedy, Emily Hayden, Kei Ouchi, Kalpana Shankar, Shan W Liu

**Affiliations:** 1 Department of Emergency Medicine, St. Marianna University Hospital, Kawasaki, JPN; 2 Department of Emergency Medicine, Brigham and Women's Hospital, Boston, USA; 3 Department of Emergency Medicine, Massachusetts General Hospital, Boston, USA; 4 Department of Psychosocial Oncology and Palliative Care, Dana-Farber Cancer Institute, Boston, USA

**Keywords:** elderly patients, fall protection, paramedic emergency medical services, telemedicine (tm), virtual observation

## Abstract

Background

Falls are a leading cause of unintentional death among adults aged 65 and above and are associated with significant injuries and healthcare costs. Older adults frequently present to the emergency department (ED) for falls. However, ED clinicians rarely focus on preventing future falls, given the lack of time and resources. We developed a novel multi-component fall prevention initiative embedded in our ED Virtual Observation Unit (VOU) Falls program.

Methods

This feasibility study, conducted at a level-1 urban teaching hospital, assessed the program’s acceptability and safety. The program included home safety evaluations, timed up and go (TUG) tests, and telemedicine reviews of medications. Data were collected using Research Electronic Data Capture (REDCap). Patient demographic data and Charlson comorbidities were recorded. Surveys adapted from the theoretical framework of acceptability (TFA) were administered to patients and providers to assess comfort, effort, fairness, acceptability, feasibility, and safety. Follow-up surveys were conducted at three months to determine changes in fall-risk behaviors. Descriptive analyses were performed to evaluate Likert scale survey data.

Results

Ultimately, 35 patients were included, with six in the intervention group and 29 in the non-intervention group. Participants felt comfortable with the ED VOU Falls program and found it acceptable, though neutral about its effectiveness in reducing fall risk. VOU physicians (22/30, 73%) found the program fair, feasible, and safe and did not interfere with ED operations or their clinical duties. At three months, no falls were reported in the intervention group, while two (8%) patients experienced falls in the control group. A higher percentage of patients in the intervention group reported actions to reduce fall risk.

Conclusion

The ED VOU Falls program appears acceptable, feasible, and safe but has a low participation rate. Further work is needed to understand provider and patient concerns regarding participation in order to adapt the program accordingly.

## Introduction

Falls are a common cause of unintentional death around the world, particularly among older adults aged 65 and older [1-3]. Falls can lead to serious injuries, health decline, social isolation, and an increased risk of admission to nursing homes [4-6]. The estimated medical cost of fall-related injuries for older adults in the United States (U.S.) is approximately $34-50 billion annually [7,8]. 

Despite the high incidence and impact of falls, emergency departments (EDs) often miss opportunities to assess and manage fall risks [9]. ED clinicians report unwillingness to spend much time on fall risk identification and management due to time pressures, ED crowding, and a potential lack of understanding regarding the implications of a fall on older adults [10]. However, interventions during or soon after an ED visit might be a window of opportunity for fall prevention [11].

To address this, we developed a novel pilot ED Virtual Observation Unit (VOU) Falls program embedded within our ED VOU [12]. The ED VOU was first created during the COVID-19 pandemic to provide safe, patient-centric care, observation level care in the home setting, and alleviate capacity challenges [13,14]. It later expanded to managing other conditions such as cellulitis, congestive heart failure, and pneumonia. We then created the ED VOU Falls program, a multi-component fall prevention initiative designed for ED patients who were at risk for falls. However, it is not known whether this novel ED VOU Falls program is feasible, acceptable, or safe. Hence, we conducted this feasibility, acceptability, and safety study of this novel ED VOU Falls program.

## Materials and methods

This feasibility, acceptability, and safety study was conducted at a level-1 urban teaching hospital in the Northeastern U.S. Our institutional review board deemed our protocol exempt. The ED VOU Falls program consisted of several key components designed to address fall risks comprehensively. First, a trained paramedic conducted a home safety evaluation, assessing potential hazards in the patient’s living environment that could contribute to falls. Second, the timed up and go (TUG) test was performed in the patient’s home by a paramedic to evaluate mobility and balance. Based on the results, the ED physician determined whether a referral to physical therapy was necessary. Finally, the review of fall-risk-increasing medications was carried out via a telemedicine consultation with an ED physician, who assessed the patient’s prescriptions for drugs that might heighten the risk of falling.

The details of our protocol and rationale for components of the program have been documented in another publication [12]. In brief, patients were enrolled between 8 am and 8 pm when a research assistant (RA) was available. Inclusion criteria consisted of a convenience sample of English-speaking patients older than 65 who were able to ambulate, deemed safe for admission to the ED VOU Falls program, and resided within the VOU catchment area. Initially, we restricted patients only to those who had presented with a fall, but then we expanded the criteria to include those older than 65 who were at risk for falls according to the Centers for Disease Control and Prevention (CDC) STEADI guidelines and who consented to participate [15]. Risk for falls was defined based on patients saying “yes” to any of three key questions: feeling unsteady when standing or walking, worrying about falling, or having fallen in the past year. Patients who resided in a healthcare facility, had active substance use or psychiatric concerns, or needed a higher level of care (e.g., needing medical admission or rehabilitation placement) were excluded. A non-intervention group of patients who had fallen or were at risk for falls consisted of those who were outside the catchment area, evaluated outside enrollment hours, or who consented only to participate in follow-up calls. Study enrollment began on March 1, 2023, with an intended duration of six months. However, the ED VOU program was discontinued on July 31, 2023, after the public health emergency waiver ended, resulting in the discontinuation of Medicare reimbursement for virtual observation care; accordingly, enrollment in the VOU Falls program ceased.

Procedures

Patients were sent home from the ED but remained part of a VOU. During this time, they could communicate with on-call VOU nurses. A community mobile integrated health (MIH) paramedic who received online VOU Falls training performed an in-home visit with patients within 24 hours and conducted (1) a home safety evaluation (see Appendix Table 5), (2) the TUG test, and (3) facilitated a telemedicine visit with the ED physician. The telemedicine ED physician reviewed these MIH components as well as any fall risk-increasing medications and subsequently referred patients to PT if patients’ TUG tests were greater than or equal to 12 seconds, as well as identified any fall risk-increasing drugs [12]. Patients were also instructed to exercise to prevent future falls.

Data collection

RAs collected patient data, including age, gender, race, insurance, primary care doctor, education, primary spoken language, and marital status, from the study site’s electronic health record system (Epic, Madison, WI). The RA also collected and determined the ED return rate for falls and non-fall complaints within three months post-ED VOU Falls program discharge in Epic [16]. To determine feasibility and acceptability, we also adapted a survey from the theoretical framework of acceptability (TFA) for patients as well as ED VOU Falls program providers. RAs called ED VOU Falls program patients one to three days after their ED VOU Falls program discharge and administered the survey. ED VOU Falls program attendings were emailed a provider-specific survey.

The RA administered the initial ED VOU Falls program follow-up and VOU attending physician questionnaire with a mix of questions using a 1-5 Likert scale and open-ended responses [17,18]. The survey questions were designed with a 5-point Likert scale (strongly disagree, disagree, no opinion, agree, and strongly agree). Based on questions adapted from the TFA, we sought to determine how the patients and ED VOU Falls program physicians rated the comfort, effort, fairness, acceptability, feasibility, and safety of the program [19]. We defined acceptability as an average of ≥4 on our 1-5 Likert scale survey questions of VOU attendings and patients (ranging from completely unacceptable to completely acceptable). We also defined safety as an average of ≥4 on our 1-5 Likert scale survey questions of attending (ranging from completely unsafe to very safe) [12]. At three months, RAs called both ED VOU Falls program patients and the non-intervention group to determine if patients changed fall-risk behaviors, such as (1) changes in home safety, (2) follow-up visit with a physical therapist if indicated, (3) changes in medications, and (4) exercise.

Study data were collected and managed using Research Electronic Data Capture (REDCap), a secure web-based software platform specifically designed to support data capture for research studies [20,21]. We aimed to recruit 50 ED VOU Falls program patients and compare them to 50 who did not participate in the program based on a sample size similar to other feasibility studies [22]. While this was a pilot study, assuming that 0.5 of the intervention group changed their fall risk behavior compared to 0.3 of the non-intervention group, we calculated needing a sample size of 96 patients to have 80% power to detect such a difference with a 95% confidence interval. This calculation aligns with estimates obtained from Statulator (https://www.statulator.com/SampleSize/ss2M.html).

Statistical analysis

Descriptive analyses were completed to compare patient demographics and discharge diagnoses of the control and intervention groups. Frequency tables were generated to present the results of both the ED VOU Falls program participants and ED VOU Falls program attending physician questionnaires. The median response was calculated for each survey question. Descriptive analyses were used to evaluate the responses to the three-month follow-up interviews.

## Results

Of the 219 patients screened, 164 were excluded, and 20 refused to participate. The primary reasons for refusal included general refusal to participate in the research (eight patients), feeling independent (one), wanting to go home immediately (one), not wanting telemedicine (one), inability to be home for the program (three), having other services (one), feeling unwell (four), and not wanting to be part of the program (one). Ultimately, 35 patients were included in the study, with six in the intervention group and 29 in the non-intervention group (Table 1). The median age was 74 years in the control group and 86 in the intervention group. Most patients in both groups were White and had an ED discharge diagnosis of fall and/or injury.

**Table 1:** Characteristics of participants

Characteristics	Non-intervention (n = 29)	Intervention (n = 6)
Age, median (interquartile range [IQR])	74 (71-77)	86 (82-90)
Sex, n (%)		
Female	14 (48.3)	4 (66.7)
Race, n (%)		
White	26 (89.7)	4 (66.7)
Black	2 (6.9)	1 (16.7)
Other	1 (3.4)	1 (16.7)
Diagnosis at discharge, n (%)		
Fall and/or injury	18 (62.1)	3 (50.0)
Pain	8 (27.6)	0 (0.0)
Other	3 (10.3)	3 (50.0)
Charlson Comorbidity Index, median (IQR)	4 (2.8-6.8)	5.5 (4.3-8.8)
Do you have insurance?		
Yes, n (%)	29 (100.0)	6 (100.0)
Do you have a primary care physician?		
Yes, n (%)	29 (100.0)	6 (100.0)
Marital status, n (%)		
Single	6 (20.7)	3 (50.0)
Married	16 (55.2)	1 (16.7)
Divorced	2 (6.9)	0 (0.0)
Widowed	5 (17.2)	2 (33.3)

ED VOU Falls program participants’ responses are presented in Table 2. The participants reported they strongly agreed that they felt comfortable with the ED VOU Falls program (Q1.1) and that the program was acceptable (Q1.8). Participants strongly disagreed that the program took significant effort (Q1.2) and that reducing fall risk interferes with other priorities (Q1.7). However, when asked if the intervention reduced their fall risk, all participants responded with “no opinion” (Q1.4), and most were neutral about whether the ED VOU Falls program clearly improved their fall risk (Q1.5) or that they felt confident in reducing their fall risk (Q1.6).

**Table 2:** Results of the questionnaire for Virtual Observation Unit (VOU) Falls program participants (n = 6)

Variables	Strongly disagree	Disagree	No opinion	Agree	Strongly agree	Median
Q1.1 I feel comfortable about the VOU Falls program, n (%)	0 (0.0)	0 (0.0)	0 (0.0)	1 (16.7)	5 (83.3)	Strongly agree
Q1.2 Participating in the VOU Falls program took effort, n (%)	5 (83.3)	1 (16.7)	0 (0.0)	0 (0.0)	0 (0.0)	Strongly disagree
Q1.3 The VOU Falls program is fair, n (%)	0 (0.0)	0 (0.0)	0 (0.0)	0 (0.0)	6 (100.0)	Strongly agree
Q1.4 The VOU Falls program has improved my fall risk, n (%)	0 (0.0)	0 (0.0)	6 (100.0)	0 (0.0)	0 (0.0)	Neutral
Q1.5 It is clear to me how the VOU Falls program will help my fall risk, n (%)	0 (0.0)	0 (0.0)	4 (66.7)	2 (33.3)	0 (0.0)	Neutral
Q1.6 I feel confident about being able to reduce my fall risk, n (%)	0 (0.0)	0 (0.0)	5 (83.3)	1 (16.7)	0 (0.0)	Neutral
Q1.7 Reducing my fall risk interferes with my other priorities, n (%)	6 (100.0)	0 (0.0)	0 (0.0)	0 (0.0)	0 (0.0)	Strongly disagree
Q1.8 The VOU Falls program was acceptable to me, n (%)	0 (0.0)	0 (0.0)	0 (0.0)	2 (33.3)	4 (66.7)	Strongly agree

Overall, ED VOU Falls program physicians reported they felt comfortable managing patients in the program, with 54.5% agreeing or strongly agreeing (Q2.1). They also reported the program was fair (62.6%, Q2.2), improves fall risk (68.2%, Q2.3), is acceptable (68.2%, Q2.7), is feasible (68.2%, Q2.8), and is safe (63.6%, Q2.9). They reported that they disagreed or strongly disagreed that reducing fall risk interferes with other ED priorities (81.8%, Q2.6). However, respondents were generally neutral about feeling confident about their ability to reduce fall risk (Q2.5) (Table 3). In terms of three-month outcomes, there were 29 participants in the control group at the beginning of the program and 25 participants in the three-month follow-up survey. None of the patients in the intervention group experienced falls, while two patients in the control group reported a fall (Table 4). A larger percentage of patients in the intervention group reported doing something to change their risk of falling compared to the non-intervention group by exercising or making their home safer. Rates of ED revisits, hospitalizations, and reported health seemed similar between the two groups. Furthermore, none of the intervention patients reported any safety issues with the ED VOU Falls program.

**Table 3:** Results of the questionnaire for Virtual Observation Unit (VOU) program attending physicians (n = 22)

Variables	Strongly disagree	Disagree	No opinion	Agree	Strongly agree	Median
Q2.1 I feel comfortable managing patients in the VOU Falls program, n (%)	1 (4.5)	2 (9.1)	7 (31.8)	9 (40.9)	3 (13.6)	Agree
Q2.2 The VOU program is fair for fall patients, n (%)	0 (0.0)	0 (0.0)	8 (36.4)	7 (31.8)	7 (31.8)	Agree
Q2.3 The VOU Falls program improves patient fall risk, n (%)	0 (0.0)	0 (0.0)	7 (31.8)	9 (40.9)	6 (27.3)	Agree
Q2.4 It is clear to me how the VOU Falls program will help patient's fall risk, n (%)	0 (0.0)	2 (9.1)	5 (22.7)	11 (50.0)	4 (18.2)	Agree
Q2.5 I feel confident about being able to reduce a patient’s fall risk, n (%)	0 (0.0)	3 (13.6)	9 (40.9)	8 (36.4)	2 (9.1)	Neutral
Q2.6 Reducing patients’ fall risk interferes with other emergency department (ED) priorities, n (%)	4 (18.2)	14 (63.6)	3 (13.6)	1 (4.5)	0 (0.0)	Disagree
Q2.7 The VOU Falls program is acceptable, n (%)	0 (0.0)	0 (0.0)	7 (31.8)	8 (36.4)	7 (31.8)	Agree
Q2.8 The VOU Falls program is feasible, n (%)	0 (0.0)	0 (0.0)	8 (36.4)	9 (40.9)	5 (22.7)	Agree
Q2.9 The VOU Falls program is safe, n (%)	0 (0.0)	0 (0.0)	8 (36.4)	10 (45.5)	4 (18.2)	Agree

**Table 4:** Results of the three-month follow-up phone interviews *Responses in each category are not mutually exclusive, meaning participants could report multiple actions or experiences.

Variables	Non-intervention (n = 25)	Intervention (n = 6)
Q3.1 Have you had a fall since your emergency department (ED) visit?		
Yes, n (%)	2 (8.0)	0 (0.0)
Q3.2 Have you done anything to change your risk of falling?		
Yes, n (%)	4 (16.0)	2 (33.3)
Q3.3 What have you done to change your risk of falling? n (%)*		
Exercise	2 (8.0)	2 (33.3)
Changed medications	0 (0.0)	0 (0.0)
Made home safer	4 (16.0)	1 (16.7)
Q3.4 Have you returned to the ED since your last visit?		
Yes, n (%)	4 (16.0)	1 (16.7)
Q3.5 Have you been admitted to the hospital since your last visit (including elective admissions)?		
Yes, n (%)	5 (20.0)	1 (16.7)
Q3.6 How has your health been since your fall? n (%)		
Better	13 (52.0)	3 (50.0)
Same	9 (36.0)	2 (33.3)
Worse	3 (12.0)	1 (16.7)
Q3.7 Any safety issues related to the Virtual Observation Unit?		
No, n (%)		6 (100.0)

## Discussion

We evaluated the feasibility and acceptability of the ED VOU Falls program and found that participants and providers felt comfortable with the program and that the providers thought it was fair, acceptable, and feasible. Furthermore, both participants and providers reported the program did not interfere with other ED priorities. Both groups were neutral about whether the ED VOU Falls program reduced fall risk.

The most notable finding in our study, however, was the low enrollment rate. We found it most surprising that it was difficult to enroll patients into the ED VOU falls program. First, many patients were initially excluded because they had not fallen recently or were being admitted to the hospital. We then changed the criteria to include at-risk for falls, which helped enrollment. However, many patients reported not wanting to be part of the research project or a fall program. One patient declined because of the telemedicine component of the program, while another felt independent. Geriatric patients may have reservations about using new technology or interventions. Studies of different fall interventions have noted that some older patients prefer to maintain their independence and may be reluctant to adopt new interventions, perceiving them as unnecessary or intrusive [23]. Additionally, the requirement to have someone visit their home may also contribute to their resistance, indicating the need for adjustments when offering the ED VOU Falls program in the future [24,25]. There is also literature that shows older adults do not feel that the quality of care received at home is on par with hospital care, so they reject it altogether [26,27].

In contrast, participants who completed the ED VOU Falls program reported no significant burden in adopting the program. However, when asked about the effectiveness of the intervention in reducing fall risk, all participants responded with “no opinion.” This lack of perceived effectiveness may be due to the preventive nature of the program, making it difficult for participants to notice immediate benefits [28]. It is also possible that patients may not have understood what fall risk actually meant, leading to a “no opinion” answer. In reality, none of the patients in the intervention group experienced recurrent falls, and a larger proportion of those in the intervention group made changes aimed at fall prevention, though due to the small sample size, we could not assess for statistical significance. Interestingly, regardless of whether they participated in the ED VOU Falls program, about half of the respondents reported an improvement in their health status since the fall. This rate was higher than the rate of lifestyle changes aimed at fall prevention, suggesting that some other unmeasured factors may be contributing to their perceived health improvement, warranting further investigation.

Regarding healthcare providers involved in the ED VOU Falls program, no significant burden was reported. Many providers believed that the program effectively contributed to fall prevention. This discrepancy between providers’ perception and participants’ perceived effectiveness might be because providers are implementing measures they believe to be effective, even if participants do not immediately recognize the benefits. The ED VOU Falls program was found to be implementable without a significant burden on both participants and attending physicians. However, the effectiveness of the program remains uncertain due to the small number of participants. Furthermore, the low enrollment rate suggests that further measures are needed to increase participation, such as motivational interviewing regarding fall risk [29].

Our study has several limitations. First, the small sample size limited the statistical power of our findings. We faced significant challenges in recruiting eligible participants within the given timeframe, which was influenced by the study design constraints and the specific inclusion criteria. Additionally, there was a notable discrepancy between the intervention (n = 6) and non-intervention (n = 29) groups, which further limited our ability to draw definitive conclusions and perform robust statistical comparisons. Second, this is not a randomized controlled trial, which may introduce selection bias and confounders, affecting the internal validity of the results. Additionally, the study employed convenience sampling, which limits its generalizability to the broader population. Third, regarding the questionnaire, neither the interviewers nor the respondents were blinded, which may have introduced social desirability bias. This could affect the external validity of the study. Fourth, part of the benefit observed in the ED VOU Falls program may be attributed to the broader ED VOU program itself. However, our fall prevention program was not feasible apart from the ED VOU, as it provided the necessary infrastructure, including paramedics and emergency medicine physicians. While this limits the ability to isolate the effects of the Falls program alone, it also highlights the importance of an integrated system for implementing such initiatives. Fifth, the term “exercise” was not specifically defined in the study, which could lead to variations in participant interpretation and affect the validity of the outcomes. Sixth, the paramedics’ assessment is currently being investigated in a separate qualitative study focusing on the perspective of the paramedics. Lastly, the most significant limitation of external validity is that most institutions currently do not have the capability to provide this type of service for their patients (i.e., home health evaluation). However, the findings show a sign of promise, suggesting that more efforts should be made to implement and test such programs in various settings.

## Conclusions

In conclusion, while the ED VOU Falls program appears acceptable, feasible, and safe to providers and those enrolled in the program, the high refusal to enroll indicates that the program needs to be adjusted to increase participation. Further work is needed to understand provider and patient concerns regarding participation in order to adapt the program accordingly.

## Appendices

**Table 5:** Home safety assessment checklist TUG TEST ≥ 12 Sec?                                          Yes            No Medication System                                           Yes            No        NA 1) Does patient have a pill box?                       Yes            No        NA 2) Do patient have expired medications mixed with unexpired ones?                            Yes            No         NA 3) Does patient have an organized medication system?                                           Yes            No         NA 4) Do you have concerns about patient’s medication system?                           Yes            No         NA

HOME SAFETY ASSESSMENT CHECKLIST			
Patient Name:		MRN:	
Paramedic Name:		Date of visit:	
Outside of home			
1. Residence building is (circle one):	Single family	Multi-family	
2. Residence building is (circle one):	Single floor	Multi-floor	
3. Sidewall and/or pathway to house is level and free from any hazards:	Yes	No	N/A
4. Driveway is free from debris/snow/ice:	Yes	No	N/A
5. Outside stairs are stable and have sturdy handrail:	Yes	No	N/A
6. Porch lights are working and provide adequate lighting:	Yes	No	N/A
7. Elevator is in working order:	Yes	No	N/A
Inside home			
1. Patient lives alone:	Yes	No	N/A
2. Patient has a pet that could potentially trip patient:	Yes	No	N/A
3. Patient has a pet/service animal that may confront visitors:	Yes	No	N/A
Livingroom			
1. Furniture is adequate height with supports to assist getting up and down:	Yes	No	N/A
2. Floor is free from clutter that would create tripping hazards:	Yes	No	N/A
3. All cords are secured and do not pose tripping hazards:	Yes	No	N/A
4. All rugs are secured to floor:	Yes	No	N/A
5. Lighting is adequate to light room:	Yes	No	N/A
6. All lighting has an easily accessible on/off switch:	Yes	No	N/A
7. Phone is readily accessible and chargeable near favorite seating areas:	Yes	No	N/A
8. Emergency numbers are available near all phones in house:	Yes	No	N/A
9. Fireplace has a cover:	Yes	No	N/A
Kitchen			
1. Items used more often are within easy reach:	Yes	No	N/A
2. Step stool is present, is sturdy, has handrail for higher shelves:	Yes	No	N/A
3. Floor mats are non-slip tread and secured to floor:	Yes	No	N/A
4. Oven controls are within easy reach:	Yes	No	N/A
5. Kitchen lighting is adequate and easy to reach switches:	Yes	No	N/A
6. ABC fire extinguisher is easily accessible:	Yes	No	N/A
Stairs			
1. Carpet is properly secured to stairs and/or all wood is properly secured:	Yes	No	N/A
2. Handrail is present and sturdy:	Yes	No	N/A
3. Stairs are free from any clutter:	Yes	No	N/A
4. Stairway is adequately lit:	Yes	No	N/A
Bathroom			
1. Tub and/or shower have a non-slip surface:	Yes	No	N/A
2. Tub and/or shower have a grab bar for stability:	Yes	No	N/A
3. Tub and/or shower is easy for patient to enter:	Yes	No	N/A
4. Toilet has nearby grab bar for assistance:	Yes	No	N/A
5. Pathway to bathroom is free from clutter and well lit:	Yes	No	N/A
Bedroom			
1. Floor is free from clutter:	Yes	No	N/A
2. Light is near bed and is easy to turn on:	Yes	No	NA
3. Phone is next to bed and within easy reach:	Yes	No	NA
4. Cell phone charger is next to bed and within easy reach:	Yes	No	NA
5. Flashlight is near bed in case of emergency:	Yes	No	NA
General			
1. Smoke detectors in all areas of the house (each floor) and tested:	Yes	No	NA
2. CO detectors on each floor of house and tested:	Yes	No	NA
3. Flashlights are handy throughout the home:	Yes	No	NA
4. Resident has medical information readily accessible:	Yes	No	NA
5. All heaters are away from flammable materials:	Yes	No	NA
Overall tips			
1. Patient has good non-skid shoes to move around house:	Yes	No	NA
2. All assisted-walking devices are readily accessible and in good condition:	Yes	No	NA
3. Home has clear pathways for wheelchair access if needed:	Yes	No	NA
4. There is a phone near the floor for ease of reach in case of a fall:	Yes	No	NA
5. All O2 tubing is less than 50 feet and is not a trip hazard:	Yes	No	NA
6. Resident has had an annual hearing and vision check:	Yes	No	NA
7. Resident has the proper hearing and visual aids in working order:	Yes	No	NA
8. All medications are properly stored and labeled with dosage times:	Yes	No	NA
For any statement marked “NO,” the following recommendations and referrals are noted below: __________________________________________________________________________________________________________________________________________________________________________			
				Signature of Resident:		Date of Assessment:	
				Signature of Paramedic:		Time of Assessment:	
